# Response Surface Methodology (RSM) Mediated Optimization of Medium Components for Mycelial Growth and Metabolites Production of *Streptomyces alfalfae* XN-04

**DOI:** 10.3390/microorganisms10091854

**Published:** 2022-09-16

**Authors:** Jing Chen, Xingjie Lan, Ruimin Jia, Lifang Hu, Yang Wang

**Affiliations:** College of Plant Protection, Northwest A & F University, Xianyang 712100, China

**Keywords:** *Streptomyces alfalfae*, antifungal activity, biomass, optimization, response surface methodology

## Abstract

*Streptomyces alfalfae* XN-04 has been reported for the production of antifungal metabolites effectively to control Fusarium wilt of cotton, caused by *Fusarium oxysporum* f. sp. *vasinfectum* (*Fov*). In this study, we used integrated statistical experimental design methods to investigate the optimized liquid fermentation medium components of XN-04, which can significantly increase the antifungal activity and biomass of XN-04. Seven variables, including soluble starch, KNO_3_, soybean cake powder, K_2_HPO_4_, MgSO_4_·7H_2_O, CaCO_3_ and FeSO_4_·7H_2_O, were identified as the best ingredients based on one-factor-at-a-time (OFAT) method. The results of Plackett–Burman Design (PBD) showed that soluble starch, soybean cake powder and K_2_HPO_4_ were the most significant variables among the seven variables. The steepest climbing experiment and response surface methodology (RSM) were performed to determine the interactions among these three variables and fine-tune the concentrations. The optimal compositions of medium were as follows: soluble starch (26.26 g/L), KNO_3_ (1.00 g/L), soybean cake powder (23.54 g/L), K_2_HPO_4_ (0.27 g/L), MgSO_4_·7H_2_O (0.50 g/L), CaCO_3_ (1.00 g/L) and FeSO_4_·7H_2_O (0.10 g/L). A verification experiment was then carried out under the optimized conditions, and the results revealed the mycelial dry weight of *S. alfalfae* XN-04 reaching 6.61 g/L. Compared with the initial medium, a 7.47-fold increase in the biomass was achieved using the optimized medium. Moreover, the active ingredient was purified from the methanol extract of *S. alfalfae* XN-04 mycelium and then identified as roflamycoin (a polyene macrolide antibiotic). The results may provide new insights into the development of *S. alfalfae* XN-04 fermentation process and the control of the Fusarium wilt of cotton and other plant diseases.

## 1. Introduction

The soil-inhabiting fungus *Fusarium oxysporum* has been reported to infect more than a hundred different crops, causing vascular wilt and even death of the plants [1]. The widely use of chemical pesticides has caused several environmental concerns such as pesticide residues and soil contamination, although it can also control the Fusarium wilt [2]. Therefore, the biocontrol of Fusarium wilt has become an environmentally friendly strategy of the management of plant diseases during the recent years [2]. Among various potential biocontrol methods, the most widely studied are beneficial microorganisms and their metabolites. As one of the most biotechnological and economic value prokaryotes, actinomycetes are responsible for the production of nearly half of the discovered secondary metabolites with economic importance [3]. Currently, more than 75% of bioactive compounds are reported from the members of *Streptomyces* [4,5,6,7,8,9,10]. Some bioactive compounds showed great activity against phytopathogenic fungi, such as kasugamycin produced by *Streptomyces kasugaensis* [11], validamycin produced by *Streptomyces hygroscopicus* [12], ningnanmycin produced by *Streptomyces noursei* [13] and wuyiencin produced by *Streptomyces albulus* var. *wuyiensis* [14].

The optimization of productive conditions is an extremely important step in the fermentation process due to their high complexity. The fermentation processes of microorganisms are influenced by many variables, including physical parameters and the nutritional compositions of culture medium [15]. The yield and metabolic profile of microorganisms are influenced by minor variations in the composition of fermentation medium [16]. The techniques used for the medium optimization of various fermentation parameters including both classical and statistical tools. Classical studies of fermentation optimization are usually performed “one-factor-at-a-time (OFAT)”, in which the level of one factor is changed while keeping the other factors constant [17]. It has the advantage of being simple and easy. However, the conventional OFAT method is tedious, time-consuming and uneconomical. In addition, when plenty of variables are involved, it ignores the combined interactions among various nutritional and physical parameters [18]. Therefore, statistical-based measures have been employed to achieve medium optimization by changing more than one variable at a time. Some of the famous statistics-based experimental designs are used in fermentation optimization, including full factorial design, fractional factorial design (FFD), Plackett–Burman design (PBD), Box–Behnken design (BBD) and central composite design (CCD) [19,20].

Response surface methodology (RSM) is a compilation of mathematical statistical techniques for designing experiments, plotting models and evaluating the effects of various variables, and establishing optimum conditions of a multivariable system to achieve significant responses [18]. This method has been extensively used for optimizing factors of fermentation medium and discovering the interactions among a multitude of fermentation parameters using a minimum of experiments [21]. In many cases, RSM uses statistically based experimental designs such as BBD and CCD to develop empirical models, which mathematically describe the relationships existing among the independent and dependent variables [22]. Further, RSM provides three-dimensional (3D) graphs and two-dimensional (2D) contour plots to clarify the shape of a response surface. Currently, RSM has been successfully applied to optimize variables for fermentation culture across a series of microorganisms including fungi, bacteria and actinomycetes in the production of industrially important metabolites [23,24,25]. For example, *Streptomyces* sp. WP-1 culture under the optimal medium designed by RSM increased fungichromin yield to 5741.7 mg/L [26]. Medium optimization using RSM contributed to a 45% (220.7 ± 5.7 mg/L) increase in rapamycin production for the *Streptomyces hygroscopicus*, compared with the unoptimized production medium (151.9 ± 22.6 mg/L) [27].

*Streptomyces alfalfae* XN-04 is a plant growth-promoting rhizobacterium (PGPR) and an excellent biocontrol strain. A previous study indicated that *S. alfalfae* XN-04 was able to produce metabolites with significant activity against some plant fungal pathogens [28]. Due to the extremely low production of biomass and long fermentation time of *S. alfalfae* XN-04, it is necessary to improve *S. alfalfae* XN-04 yield in submerged fermentation. Moreover, the main antifungal ingredient in the metabolites of *S. alfalfae* XN-04 remains unclear. In this study, we assessed the following objectives: (1) optimize the fermentation medium of *S. alfalfae* XN-04 for the maximum biomass at the level of flask fermentation; (2) purify and identify the main antifungal ingredient of *S. alfalfae* XN-04 using various chromatographic and spectroscopic methods.

## 2. Materials and Methods

### 2.1. Microorganisms and Culture Conditions

*S. alfalfae* XN-04 was originally isolated from the rhizosphere soil sample collected from Qinghai Province, China [28]. The strain XN-04 was cultured on mannitol soybean agar (MS) and incubated at 28 °C for 14 days to produce spores. The phytopathogenic fungus *Fusarium oxysporum* f. sp. *vasinfectum* (*Fov*) was preserved in the Biological Control of Plant Disease Laboratory of Northwest Agriculture and Forestry University. The fungus was cultured on potato dextrose agar (PDA) plates and incubated at 28 °C for 7 days.

### 2.2. Inoculum Preparation

Inoculum was prepared by inoculating 5 spore cakes (6 mm) of *S. alfalfae* XN-04 into a 250 mL Erlenmeyer flasks containing 100 mL Gauze’s synthetic No. 1 (GS) medium. Then, the flasks were incubated at 28 °C on a shaker at 180 rpm for 3 days. The inoculum quantity was controlled at 5% (*v*/*v*) in all the fermentation experiments.

### 2.3. Antifungal Activity Assay

Fermentation culture was centrifuged at 5000× *g* rpm at 4 °C for 10 min, and the precipitate was collected. Three volumes of methanol (MeOH) were added, and the sample was then processed with ultrasonication for 10 min. After filtration using filter paper, the MeOH extract was evaporated to dryness using a rotary vacuum evaporator under reduced pressure at 50 °C. The MeOH extract was redissolved in MeOH to prepare a stock solution with a final concentration of 50 mg/mL. The antifungal activity was estimated with the method of mycelium growth in sealed plates described by previous study [29]. Briefly, 50 μL of the MeOH extract and 50 mL of sterile PDA medium were uniformly mixed and poured into three culture dishes (9 cm in diameter). Inverted mycelial plugs (6 mm in diameter) taken from the active periphery of 7-day-old fungus colonies were placed on the center of these plates. Plates with an appropriate volume of MeOH were used as control. Plates were incubated for 7 days in a growth chamber at 28 °C, and the colony diameter was measured. Each treatment had three plates, and the experiment was repeated three times. The levels of inhibition were calculated by using the equation as follows:

Inhibition rate (%) = (the colony diameter of control − the colony diameter of treatment)/(the colony diameter of control) × 100(1)

### 2.4. Single-Factor Optimization of Fermentation Medium Composition

Single-factor experiments were used to screen the most significant medium substrate. GS medium was used as the initial medium. A total of 9 different carbon sources and 9 different nitrogen sources to replace corresponding carbon and nitrogen source in the initial medium while other compositions were kept constant at their original concentration. Glucose, sucrose, xylose, galactose, fructose, millet flour, rice flour, corn steep liquor and soluble starch (at 20 g/L) were investigated as carbon sources. The influence of KNO_3_, (NH_4_)_2_SO_4_, NH_4_NO_3_, NaNO_3_, urea, yeast extract, beef extract, soybean cake powder and peptone (at 1 g/L) as nitrogen sources were simultaneously investigated. Similarly, K_2_HPO_4_, MgSO_4_·7H_2_O, CaCO_3_, KCl, KH_2_PO_4_ and NaCl (at 0.1 g/L) were selected to optimize mineral salts. Additionally, CuSO_4_·5H_2_O, CoCl_2_·6H_2_O, MnCl_2_·4H_2_O, FeSO_4_·7H_2_O and ZnSO_4_·7H_2_O (at 0.01 g/L) were chosen as trace elements. At the end of fermentation, samples obtained from fermentation culture were used to calculate mycelium dry weight and antifungal activity.

### 2.5. Single-Factor Concentration Screening Test

In order to select the most significant concentration by using the single variable procedure, soluble starch (0, 20, 40, 60, 80 and 100 g/L), KNO_3_ (0, 1, 2, 3, 4 and 5 g/L), soybean cake powder (0, 20, 40, 60, 80 and 100 g/L), K_2_HPO_4_ (0, 0.2, 0.4, 0.6, 0.8 and 1.0 g/L), MgSO_4_·7H_2_O (0, 0.2, 0.4, 0.6, 0.8 and 1.0 g/L), CaCO_3_ (0, 0.2, 0.4, 0.6, 0.8 and 1.0 g/L) and FeSO_4_·7H_2_O (0, 0.02, 0.04, 0.06, 0.08 and 0.1 g/L) were added to the initial medium, respectively. The mycelium dry weight and antifungal activity of samples were determined as described above.

### 2.6. Plackett–Burman Design (PBD)

The Plackett–Burman design (PBD) is an effective method to investigate the effect of medium composition, and is very helpful for screening the most important variables with respect to their main effects. The results of PBD do not describe the interaction among these variables but it is used to screen and evaluate the variables that have a significant impact on response. The total number of experiments performed according to PBD is *n* + 1, where *n* is the number of variables. In this study, a range of 12 experiments were constructed using the Design-Expert software version 8.0.6.1 (Stat-Ease, lnc., Minneapolis, MN, USA) for 7 different independent variables including soluble starch, KNO_3_, soybean cake powder, K_2_HPO_4_, MgSO_4_·7H_2_O, CaCO_3_ and FeSO_4_·7H_2_O. Each independent variable was tested at 2 levels, high and low, denoted by (+) and (−), respectively (Table 1). A total of 4 dummy variables were designed in these 12 experiments to calculate the standard error. All tests were performed in triplicate, and the average of mycelium dry weight was treated as responses. The effect of medium components on mycelium dry weight was determined by *p*-values obtained by analysis of variance (ANOVA). A *p*-value (Prob > F) of less than 0.05 to indicate when factors are mathematically significant.

### 2.7. The Steepest Ascent Experiment

The steepest ascent experiment was designed to determine a suitable direction according to the results of PBD, and the change step was determined according to the effect value of each factor. The direction of the steepest ascent experiment and the step of change were determined by the three main influencing factors of soluble starch, soybean cake powder and K_2_HPO_4_ according to the results of PBD, and the area of mycelial dry weight maximum production could be approached rapidly.

### 2.8. Box–Behnken Design (BBD)

Response surface methodology (RSM) based on Box–Behnken design (BBD) was used to optimize optimal levels of medium compositions. Based on the trajectory of the steepest ascent experiments, 3 variables were selected and their concentrations were arranged at 17 levels, with 5 replicates at the center point to study their interaction effect. According to the design, 3 variables with the highest confidence levels were prescribed at 3 levels, coded −1, 0 and +1 for low, middle and high concentrations respectively (Table 2). In order to predicting the maximum value of response, a ternary quadratic equation was established to correlate the relationship between variables and response. The validity of this model was determined based on Student’s *t* test. Data obtained from the BBD were subjected to first and second order multiple regression analysis using the method of least squares to obtain the parameters of the mathematical models. Model coefficients, R_2_ values, *F* values and significance probabilities generated by the Design-Expert software version 8.0.6.1 (Stat-Ease, lnc., Minneapolis, MN, USA) provided a confirmation of the significance of each experimental variable. The optimal medium composition for improving the biomass of *S. alfalfae* XN-04 were obtained by solving this ternary quadratic equation and by analyzing the response surface contour plots. Combined with the regression equation, the 3D response surfaces and 2D contour plots were plotted by the Design-Expert software to understand the interaction effects of medium components and optimum concentration of each variable required for maximum biomass production. The 2D contour plot is used to determine the interaction strength between the two variables according to the radius of the curved surface of the arc [30]. The elliptic order of contour indicates that the interactions between corresponding variables were significant, while the circular order reveals non-significant interactions [30].

In order to determine the accuracy of this model and verify the results, an experiment under the optimal conditions obtained from BBD was performed, followed by comparing the response value with the predicted data.

### 2.9. Purification and Identification of Antifungal Compounds

*S. alfalfae* XN-04 was inoculated into the optimized medium and incubated at 28 °C with shaking at 180 rpm for 14 days. After incubation, a total of 7.5 L fermentation broth was centrifuged at 5000× *g* rpm at 4 °C for 10 min, and the precipitate was collected. Three volumes of MeOH were added, and the sample was then processed with ultrasonication for 10 min. After filtration using filter paper, the MeOH extract was evaporated to dryness using a rotary vacuum evaporator under reduced pressure at 50 °C to yield a brown crude extract (13.2 g). The crude extract was purified on silica gel column chromatography (300 mesh, Qingdao Marine Chemical Inc., Qingdao, China) and subjected to gradient elution with a mixture solvent consisting of dichloromethane/MeOH from 10:1 to 2:1 (*v*/*v*) to obtain 10 fractions (Frs. 1–10). The paper disk (6 mm in diameter) diffusion assay was used to evaluate the antifungal activities of each fraction [31]. Further separation of Fr. 5 (672 mg) by semi-preparative HPLC on a C18 column using acetonitrile/water (45/55, *v*/*v*) as the mobile phase with a flow rate of 10 mL/min, collected the peak at 10.08 min (UV detector 363 nm) to yield compound 1 (220 mg, yellow amorphous solid).

The antifungal compound 1 was analyzed by high performance liquid chromatography-tandem mass spectrometry (HPLC-MS/MS; LC-30A + TripleTOF5600+, AB SCIEX, Framingham, MA, USA). Mass spectrometry (MS) and tandem mass spectrometry (MS/MS) were performed using electrospray ionization (ESI) detection in the positive mode. Nuclear magnetic resonance (NMR) spectra were recorded on a Bruker Avance III instrument at 600 MHz to elucidate the structure of compound **1**.

### 2.10. Statistical Analysis

All experiments were repeated three times independently and the summary statistics are expressed as mean ± standard deviation (SD). Data were analyzed by one-way ANOVA with Duncan’s post hoc pairwise multiple range test. Differences between sample mean values of *p* < 0.05 were considered to be significant. Data obtained from the PBD and BBD were analyzed with a statistical software package Design-Expert software (Version 8.0.6.1, Stat-Ease Inc., Minneapolis, MN, USA).

## 3. Results

### 3.1. Effect of Different Nutrient Sources on Antifungal Metabolites Production and Biomass

**Figure 1:**
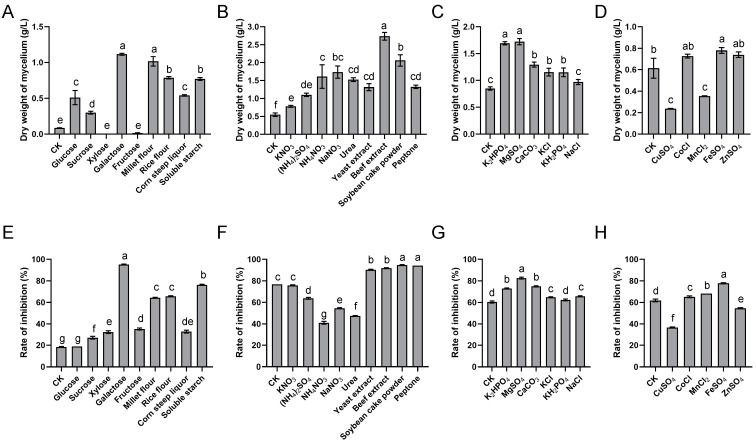
Effect of different nutrient sources on the biomass yield and antifungal metabolites production of *S. alfalfae* XN-04. (**A**,**E**) Effect of carbon sources. (**B**,**F**) Effect of nitrogen sources. (**C**,**G**) Effect of mineral salts. (**D**,**H**) Effect of trace elements. The dry weight of mycelium was used as indication of biomass, and the rate of inhibition was used as indication of antifungal metabolites production. Bars represent the SD of three replicates. Different lowercase letters indicate a significant difference at *p* < 0.05 level by Duncan’ s new multiple range test.

The results showed that different nutrient sources had significant effects on the cell growth and antifungal activity of *S. alfalfae* XN-04 (Figure 1).

#### 3.1.1. Carbon Sources

**Figure 2:**
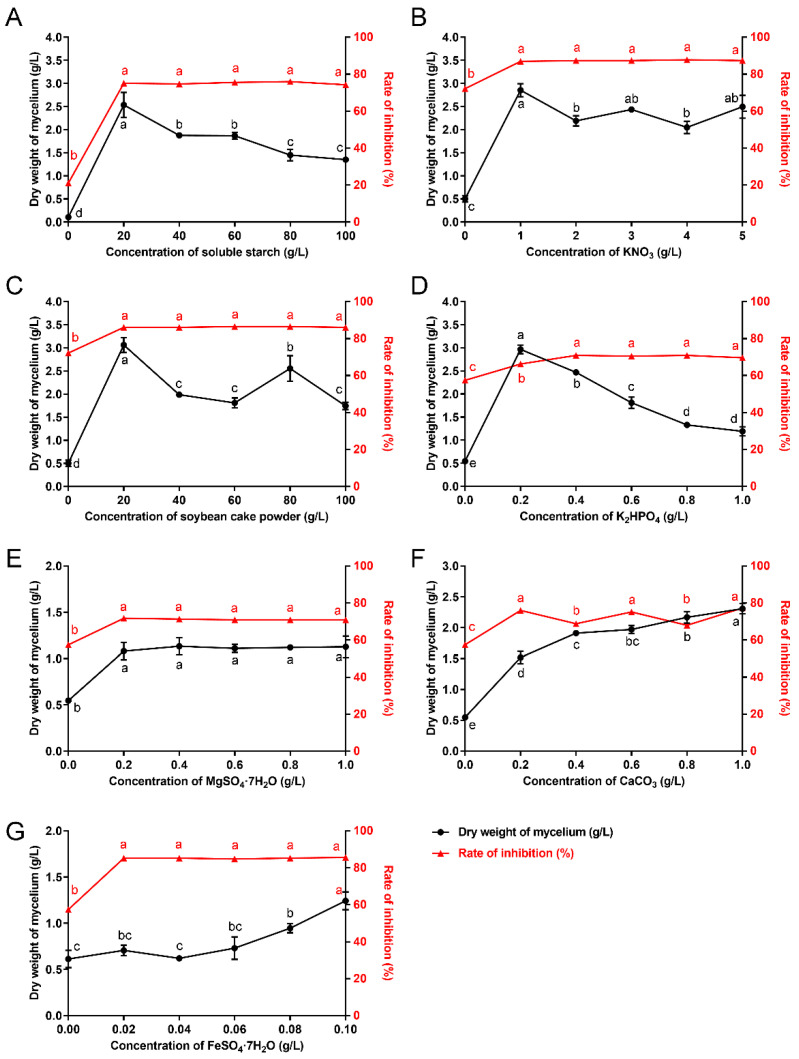
Effect of nutrient sources at different concentrations on the biomass yield and antifungal metabolites production of *S. alfalfae* XN-04. (**A**) Soluble starch. (**B**) KNO_3_. (**C**) Soybean cake powder. (**D**) K_2_HPO_4_. (**E**) MgSO_4_·7H_2_O. (**F**) CaCO_3_. (**G**) FeSO_4_·7H_2_O. The dry weight of mycelium was used as indication of biomass (corresponds to the left X axis), and the rate of inhibition was used as indication of antifungal metabolites production (corresponds to the right Y axis). Bars represent the SD of three replicates. Different lowercase letters indicate a significant difference at *p* < 0.05 level by Duncan’ s new multiple range test.

The fermentation medium was supplemented with 20 g/L of different carbon sources to determine their individual influence. The results revealed that the maximum biomass was obtained with galactose (1.12 g/L), followed by millet flour (1.02 g/L) (Figure 1A). Rate of inhibition (%) was used to indicate the production of antifungal metabolites. Maximum inhibition rate was achieved with galactose (95.5%), followed by soluble starch (76.33%) (Figure 1E). Notably, the results showed that the fermentation medium containing millet flour and rice flour increased the levels of biomass but these agents were less efficient in the production of antifungal metabolites (Figure 1A,E). Although maximum biomass and antifungal metabolites was produced by galactose, soluble starch was selected for further studies due to cost consideration. The results of concentration screening test showed that the maximum biomass was produced at the 20 g/L soluble starch level (Figure 2A). However, the antifungal activity remained unchanged between the concentrations of 20 g/L and 100 g/L (Figure 2A). This means the highest antifungal metabolites production was also obtained at the 20 g/L soluble starch level. Therefore, 20 g/L soluble starch was chosen as the carbon source for further experiments.

#### 3.1.2. Nitrogen Sources

Each organic and inorganic nitrogen source supported growth (Figure 1B). The results showed that the maximum biomass was obtained with beef extract (2.74 g/L), followed by soybean cake powder (2.06 g/L) (Figure 1B). Maximum inhibition rate was achieved with soybean cake powder (94.68%), followed by peptone (94.2%) (Figure 1F). Generally, a higher mycelial growth inhibition rate was achieved when the complex organic nitrogen source was added in fermentation medium (Figure 1F). Notably, the production of antifungal metabolites was negatively affected by nitrogen sources favorable for growth, such as NH_4_NO_3_, NaNO_3_ and urea (Figure 1F). Although the maximum biomass was obtained by beef extract, soybean cake powder was selected for further studies due to cost considerations. Additionally, considering the ratio of organic nitrogen sources to inorganic nitrogen sources, KNO_3_ was selected as the inorganic nitrogen source. Therefore, KNO_3_ at 1 g/L and soybean cake powder at 20 g/L were the most suitable nitrogen sources for maximum biomass (Figure 2B,C).

#### 3.1.3. Mineral Salt

Comparative studies on suitable mineral salts were carried out with results shown in Figure 1C,G. The results revealed that K_2_HPO_4_, MgSO_4_·7H_2_O and CaCO_3_ had distinct positive effects both on cell growth and antifungal metabolites production of *S. alfalfae* XN-04. The results of concentration screening test showed that the optimum concentrations of K_2_HPO_4_, MgSO_4_·7H_2_O and CaCO_3_ were 0.2 g/L, 0.4 g/L and 1.0 g/L, respectively (Figure 2D–F).

#### 3.1.4. Trace Elements

The results showed that the maximum biomass and inhibition rate was achieved with FeSO_4_·7H_2_O (at 0.1 g/L), and was therefore, selected for further studies (Figure 1D,H and Figure 2G).

Taking all this into account, seven factors (soluble starch, KNO_3_, soybean cake powder, K_2_HPO_4_, MgSO_4_·7H_2_O, CaCO_3_ and FeSO_4_·7H_2_O) were chosen for further optimization and their initial concentrations were determined.

### 3.2. Plackett–Burman Design (PBD)

To evaluate the most significant variables for biomass accumulation, PBD design was construed. A total of 12 different medium were prepared and the experimental runs were carried out based on the experimental matrix of PBD, and the observed responses are shown in Table 3. There was a variation from 2.33 to 4.75 g/L in mycelium dry weight (Table 3).

Regression analysis and ANOVA of the PBD were performed and represented in Table 4. The results showed that the regression was significant (*p*-value 0.0195), with the *p*-values for soluble starch, soybean cake powder and K_2_HPO_4_ were 0.0055, 0.0204 and 0.0261, respectively (Table 4). Values of prob > *F* less than 0.0500 indicated the model term was significant, and in this case X_1_, X_2_ and X_4_ were significant model terms. Therefore, they were considered to be the most significant variables that affected mycelial dry weight. The other variables had small effects and low confidence levels (*p* > 0.05) which were considered mathematically insignificant (Table 4). The results of PBD also clearly showed that soluble starch, soybean cake powder and K_2_HPO_4_ had coefficient estimates of 0.46, −0.31 and 0.29, respectively, which meant that soluble starch and K_2_HPO_4_ exhibited a positive effect, while soybean cake powder acted negatively (Table 4). According to these results, X_1_, X_2_ and X_4_ were selected for further optimization. The next step in the optimization should increase the concentration of soluble starch and K_2_HPO_4_ in the fermentation medium, and reduce the concentration of soybean cake powder.

### 3.3. The Steepest Ascent Experiment

In order to further approach the maximum response value region for each main factor for subsequent response surface analysis, the design of the steepest ascent test was performed. In the steepest ascent experiment, the change range of the concentrations of soluble starch, soybean cake powder and K_2_HPO_4_ were selected as 1.5 g/L, 1.5 g/L and 0.015 g/L (Table 5). All other components were fixed at those combinations of the maximum concentration of mycelial dry weight according to the results obtained from PDB (Run 4). The steepest ascent test design and its response values are shown in Table 5. The results revealed that the mycelial dry weight showed a trend of increasing and then decreasing, indicating that the test design was reliable. When the 2nd experimental was run, the mycelial dry weight reached the maximum (5.31 g/L), which was the region of the maximum response value of the three factors. Therefore, this point was chosen to set up basal concentrations for BBD.

### 3.4. Box–Behnken Design (BBD)

To further increase mycelial dry weight, the interactive effects of the most important variables, i.e., soluble starch (X_1_), soybean cake powder (X_3_) and K_2_HPO_4_ (X_4_), were examined by RSM using BBD. The experimental design and the corresponding results are described in Table 6. The response (Y) fits with the ternary quadratic equation (final equation in terms of coded factors):

Y= +5.99 − 0.54 × X_1_ + 0.081 × X_3_ + 0.66 × X_4_ + 0.71 × X_1_X_3_ + 0.93 × X_1_X_4_ + 0.58 × X_3_X_4_ − 9.92 × X_12_ − 2.05 × X_32_ − 1.05X_42_ (2)

Table 6 showed the predicted responses of BBD on the basis of the above equation. This model equation’s importance was statistically evaluated by the F test for the ANOVA, and the results were predicted in Table 7. The ANOVA regression model demonstrated a determination coefficient (R_2_) of 0.9926, which meant 99.26% variability in the response can be explained by this model (Table 7). The value of the adjusted determination coefficient (R_2adj_) was 0.9831 (Table 7). The higher value of R_2adj_ showed the strong significance of the model. R_2_ and R_2adj_ were in close agreement. Moreover, a low value of coefficient of variation (5.11%) indicated the reliability and precision of these experiments executed (Table 7). An adequate precision value (26.776) measured the signal-to-noise ratio, and a ratio > 4.0 was desirable (Table 7). In this model, the very low *p*-value (<0.0001) for biomass is lower than 0.05, which revealed that the quadratic model we established was significant (Table 7). Based on the results of ANOVA table, it was found that the factors X_1_, X_4_, X_1_X_3_, X_1_X_4_, X_3_X_4_, X_12_, X_32_ and X_42_ were all significant model terms (Table 7). The value of lack-of-fit was not significant (*p* > 0.05) for this model which suggested that the experimental data were in solid agreement with predicted responses (Table 7).

**Figure 3:**
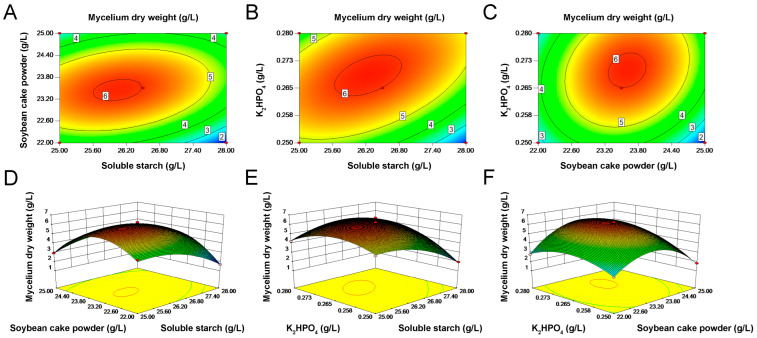
Contour plot described by the model on mycelial dry weight. (**A**) Effect of soluble starch and soybean cake powder. (**B**) Effect of soluble starch and K_2_HPO_4_. (**C**) Effect of soybean cake powder and K_2_HPO_4_. Response–surface curve of mycelial dry weight showing mutual interactions. (**D**) Soluble starch and soybean cake powder. (**E**) Soluble starch and K_2_HPO_4_. (**F**) Soybean cake powder and K_2_HPO_4_. Other variables, except for the two in each figure, were maintained at zero level in coded units. The numbers 2–6 in the figure indicate the mycelium dry weight at this location.

The 3D response surface and the 2D contour plots are generally the graphical representations of the regression equation. The elliptic order of contour in Figure 3A suggested that the interaction between soluble starch and soybean cake powder was significant. Similarly, there was a strong interaction between soluble starch and K_2_HPO_4_ (Figure 3B). Moreover, the circular order of contour of Figure 3C suggested that the interaction between soybean cake powder and K_2_HPO_4_ had a less significant effect. The 3D response surface aided in the visual determination of optimum levels of each variable as they interact. Three-dimensional response surface plots depicted mycelial dry weight with respect to soluble starch versus soybean cake powder (Figure 3D). Due to the interaction response of soluble starch with soybean cake powder, biomass increased with the increasing concentration of soluble starch and soybean cake powder up to 26.26 g/L and 23.54 g/L, respectively (Figure 3D). Further continuous increases in their concentrations resulted in a decrease in biomass. Figure 3E represented the interaction effect of soluble starch and K_2_HPO_4_ on mycelial dry weight. With an increase in soluble starch (25–26.26 g/L) and K_2_HPO_4_ (0.25–0.269 g/L) concentration, the biomass increased, and then dropped. Figure 3F revealed that the maximum mycelial dry weight was produced at a high level of soybean cake powder (26.26 g/L) and K_2_HPO_4_ (0.269 g/L) in the design range.

### 3.5. Validation of the Optimized Medium

**Figure 4:**
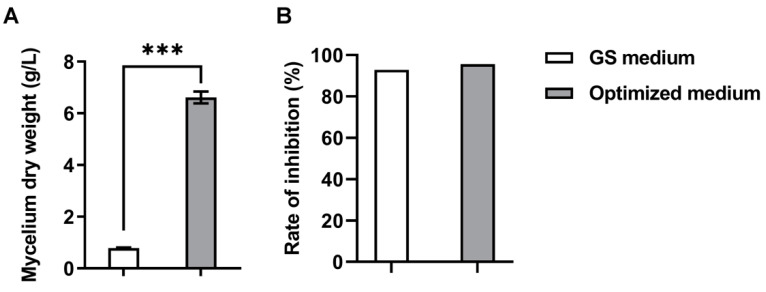
Validation of statistical optimization medium and the initial medium (GS medium). (**A**) Mycelial dry weight. (**B**) Rate of inhibition. Bars represent the SD of three replicates. *** indicates statistical significance based on the two-tailed test (*p* < 0.0001).

Based on the results of optimized medium composition, the maximum mycelial dry weight of *S. alfalfae* XN-04 was predicted to be 6.12 g/L when the concentrations of X_1_, X_3_ and X_4_ were 26.26 g/L, 23.54 g/L and 0.269 g/L, respectively. The result predicted by RSM was validated by carrying out an experiment using best-predicted solutions for growth of *S. alfalfae* XN-04. Under the optimized conditions, the average mycelial dry weight of *S. alfalfae* XN-04 reached 6.61 g/L, which was close to the RSM predicted value, suggesting that the experimental and the predicted values were in good agreement (Figure 4A). Compared with GS medium, the optimized medium enhanced mycelial dry weight by 7.47-fold (Figure 4A). Furthermore, under optimal conditions, the methanol extracts of cells show a stronger suppressive effect against *Fov* (95.65%) (Figure 4B). Therefore, we concluded that the model established in this paper was accurate and reliable for predicting the biomass of *S. alfalfae* XN-04.

### 3.6. Purification and Structural Elucidation of the Antifungal Compound

**Figure 5:**
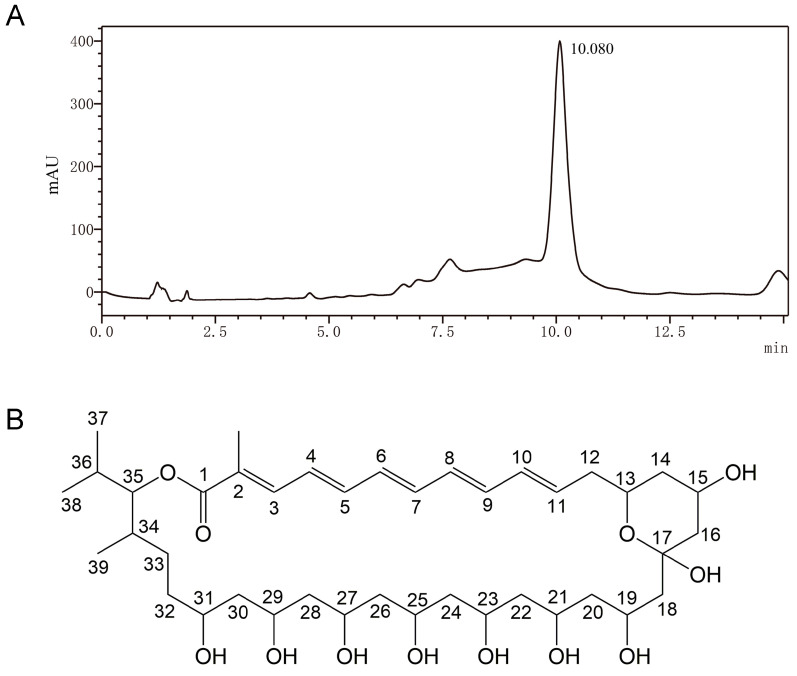
HPLC analysis and structure of roflamycoin produced by *S. alfalfae* XN-04. (**A**) HPLC analysis of Fr. 5 at 363 nm. (**B**) The structure of roflamycoin produced by *S. alfalfae* XN-04.

A series of chromatographic procedures were performed to purify the main antifungal ingredient in the MeOH extract of *S. alfalfae* XN-04. A total of 10 fractions were obtained from the silica gel column chromatography (Figure S1), among which Fr. 5–9 exhibited antifungal activity (Table S1). Fr. 5 demonstrated strong antifungal activity, producing a radius of inhibition zone > 14 mm. Therefore, Fr. 5 was subjected to semi-preparative HPLC for further purification. The chromatogram was monitored at 363 nm, and a peak (compound **1**) was observed at the retention times of 10.08 (Figure 5A). Finally, compound **1** was obtained as a yellow amorphous solid. The results of LC-MS/MS showed that the most abundant high mass ion [M + H]+ was found at *m*/*z* 739.4619 (Figure S2). Compound **1** was identified as roflamycoin (molecular weight, 738; molecular formula, C_40_H_66_O_12_) by comparing its NMR data (Table S1) with the previous study [32].

## 4. Discussion

Recent studies reported that actinomycetes, particularly *Streptomyces* spp., acted as effective biocontrol agents against multiple phytopathogens [33]. *Streptomyces* spp. can directly or indirectly benefit plants due to its ability to produce antibiotics, hydrolytic enzymes, or enzyme inhibitors [34]. A previous study indicated that *S. alfalfae* XN-04 can colonize the cotton plant root system, inhibiting the mycelial growth of *Fov* by production of hydrolytic enzymes and antifungal secondary metabolites [28]. However, the extremely low yield of antifungal metabolites and long fermentation period of *S. alfalfae* XN-04 have restricted its further research and market applications. In order to maximize the yield of antifungal metabolites and to provide data to support the large-scale and low-cost expansion of *S. alfalfae* XN-04, this study was conducted to optimize the fermentation medium components of *S. alfalfae* XN-04. Additionally, an antifungal compound was purified from the metabolites of *S. alfalfae* XN-04 and was identified as roflamycoin.

Carbon sources are essential components in constructing cellular materials and are also used as energy sources [35]. Nitrogen is an important element for nucleic acid and protein, which are the raw materials synthesized by microorganisms to create cellular metabolites [36]. In the process of microorganism fermentation, interaction between cell growth and secondary metabolites secretion is critically influenced by the growth-limiting nutrient at certain concentrations [37]. Therefore, selection as well as optimization of nutritional components is an indispensable step in large-scale production of probiotic biomass at a reasonable cost. Generally, secondary metabolites production in *Streptomyces* spp. is often stimulated by slowly assimilated complex carbohydrates (e.g., soluble starch, dextrins), but is suppressed by rapidly utilized carbon sources (e.g., glucose). Shakeel et al. (2016) found that soluble starch was the best carbon for the cell growth of *Streptomyces platensis* 3–10 [37]. Jacob et al. (2014) reported that *Streptomyces nogalater* NIIST A30 was able to produce antibacterial metabolites at a higher level with starch as carbon source [38]. In this study, the results showed that the biomass and antifungal metabolites production of *S. alfalfae* XN-04 were significantly improved with the supplementation of soluble starch and soybean cake powder (Figure 1A,B,E,F). Moreover, the results also revealed that glucose had negative effect on the antifungal metabolites production of *S. alfalfae* XN-04 (Figure 1E). These findings appear to be consistent with the results in related previous studies [39]. A possible explanation of this phenomenon is that glucose may cause catabolite repression, in which the production of enzymes for secondary metabolites biosynthesis might be inhibited [40].

Minerals such as K^+^, Ca^2+^, Na^+^ and Mg^2+^ have been known to play a crucial role in the metabolic process of all microorganisms, as it is essential for the formation of cell mass and acts as a cofactor for many biosynthetic enzymes to catalyze the necessary reactions [35]. Zhu et al. (2007) made an effort to ameliorate the production of avilamycin using *Streptomyces viridochromogenes* Tu57–1 and by adopting BBD in which they found a maximum production of 88.33 mg/L (2.80-fold increase) in the optimized medium with the ingredients such as MgSO_4_·7H_2_O 0.37 g/L, CaCl_2_·2H_2_O 0.39 g/L, soybean flour 21.97 g/L and soluble starch 37.22 g/L [41]. Peng et al. (2020) reported that the fungichromin production in *Streptomyces* sp. WP-1 was markedly increased in medium supplemented with magnesium phosphate or calcium phosphate [26]. In this study, the results showed that K_2_HPO_4_, MgSO_4_·7H_2_O and CaCO_3_ had distinct positive effects both on microorganism growth and on antifungal metabolites production by *S. alfalfae* XN-04 (Figure 1C,G). Moreover, the results also revealed that the demand for Ca^2+^ of *S. alfalfae* XN-04 was at a high level (Figure 2F). This finding suggests that Ca^2+^ is a key factor in *S. alfalfae* XN-04 growth. This finding appears to be consistent with the results in related previous studies. A previous study reported that there was a high calcium content in spores of *Streptomyces* spp. and that for some species it was an essential triggering factor of spore germination [42]. Additionally, CaCO_3_ used as medium substrate can balance the pH of the fermentation broth by reacting with the acid, which was produced by the process of microorganism fermentation to form neutral salts and CO_2_, the latter escaping from the medium.

In order to clarify the interactions among factors selected by single-factor experiments, further work will be needed on mixing conditions. Generally, in non-growth associated fermentation, secondary metabolites production was substantially proportional to the quantity of biomass [18]. Therefore, it is essential to increase the total biomass of microorganisms, especially for *S. alfalfae* XN-04, since its antifungal metabolites were produced inside the cells. Taking in account the above theory, we focused on optimization of medium components, seeking a substantial increase in biomass without reducing the production of secondary metabolites. Therefore, mycelial dry weight was utilized as the response in subsequent experiment designs.

Statistical experimental design was an efficient method for optimizing the effects of each variable. The PBD enables screening for the most significant variables of microorganism fermentation, and that the steepest ascent experiment made the result approximate to the optimal region for easily carrying out the subsequent experiments. Finally, the RSM detected the optimum levels of significant factors. In 2004, Elibol tried to optimize a fermentation medium using a 24 full factorial CCD for the production of benzoisochromanequinone polyketide antibiotic actinorhodin with *Strptomyces coelicolor* A3 (2), where 200 mg/L of antibiotic yield was attained under the optimized medium composition, which was 32% higher than that of the unoptimized medium (148 mg/L) and was very close to the predicted yield (195 mg/L) [43]. The maximization of olivanic acid production using *Streptomyces olivaceus* MTCC 6820 in shake flask was executed in which 415 mg/L of yield was achieved in the medium optimized with CCD and it was eight times higher when compared with the normal unoptimized medium (50 mg/L) [44]. The results were in agreement with these results. In this study, the mycelial dry weight of *S. alfalfae* XN-04 reached 6.61 g/L (7.47-fold increase) in the medium optimized with RSM.

In conclusion, this study optimized the fermentation medium compositions for biomass yield and antifungal metabolites production of *S. alfalfae* XN-04. This study also reported an antifungal secondary metabolite, roflamycoin, which was purified from *S. alfalfae* XN-04 metabolites.

## Figures and Tables

**Table 1:** The level and code of variables chosen for PBD.

Name	Factors	Code Value (g/L)	
		−1	+1
X_1_	Soluble starch	20	25
X_2_	KNO_3_	1.00	1.25
X_3_	Soybean cake powder	20	25
X_4_	K_2_HPO_4_	0.20	0.25
X_5_	MgSO_4_·7H_2_O	0.40	0.50
X_6_	CaCO_3_	1.00	1.25
X_7_	FeSO_4_·7H_2_O	0.100	0.125

**Table 2:** The level and code of variables chosen for BBD.

Name	Factors	Code Value (g/L)		
		−1	0	+1
X_1_	Soluble starch	25.00	26.50	28.00
X_3_	Soybean cake powder	22.00	23.50	25.00
X_4_	K_2_HPO_4_	0.250	0.265	0.280

**Table 3:** The design and results of PBD.

Run	Code Number							Mycelial Dry Weight (g/L)
	X_1_	X_2_	X_3_	X_4_	X_5_	X_6_	X_7_	
1	−1	−1	−1	1	−1	1	1	3.39
2	1	1	1	−1	−1	−1	1	2.75
3	−1	−1	1	−1	1	1	−1	2.52
4	1	−1	1	1	1	−1	−1	4.75
5	1	1	−1	1	1	1	−1	4.54
6	1	1	−1	−1	−1	1	−1	3.73
7	−1	1	1	1	−1	−1	−1	3.14
8	1	−1	−1	−1	1	−1	1	4.34
9	1	−1	1	1	−1	1	1	3.54
10	−1	1	−1	1	1	−1	1	3.28
11	−1	1	1	−1	1	1	1	2.33
12	−1	−1	−1	−1	−1	−1	−1	3.50

**Table 4:** Analysis of variance of PBD.

Source	Factor	Freedom	Coefficient Estimate	Sum of Squares	Mean Square	*F* Value	Prob > *F*
Model		7	3.48	6.15	0.88	10.41	0.0195 *
X_1_	Soluble starch	1	0.46	2.51	2.51	29.77	0.0055 **
X_2_	KNO_3_	1	−0.19	0.43	0.43	5.09	0.0871
X_3_	Soybean cake powder	1	−0.31	1.17	1.17	13.89	0.0204 *
X_4_	K_2_HPO_4_	1	0.29	1.00	1.00	11.89	0.0261 *
X_5_	MgSO_4_·7H_2_O	1	0.14	0.24	0.24	2.89	0.1645
X_6_	CaCO_3_	1	−0.14	0.24	0.24	2.89	0.1645
X_7_	FeSO_4_·7H_2_O	1	−0.21	0.54	0.54	6.42	0.0644
R^2^ = 0.9479							

* indicates a significant difference at *p* < 0.05 level; ** indicates a significant difference at *p* < 0.01 level.

**Table 5:** The design and results of the steepest ascent experiment.

Run	X_1_ (g/L)	X_3_ (g/L)	X_4_ (g/L)	Mycelial Dry Weight (g/L)
1	25.00	25.00	0.250	4.42
2	26.50	23.50	0.265	5.31
3	28.00	22.00	0.280	5.06
4	29.50	20.50	0.295	4.99
5	31.00	19.00	0.310	4.27
6	32.50	17.50	0.325	4.07
7	34.00	16.00	0.340	3.73

**Table 6:** The design and results of BBD.

Run	Code Number			Actual Value (g/L)	Predicted Value (g/L)
	X_1_	X_3_	X_4_		
1	0	0	0	6.25	5.99
2	0	1	1	4.26	4.20
3	0	0	0	5.89	5.99
4	0	0	0	5.81	5.99
5	−1	−1	0	4.35	4.19
6	1	0	1	5.17	5.07
7	0	1	−1	1.82	1.73
8	−1	0	−1	5.17	5.07
9	0	−1	1	2.79	2.88
10	0	0	0	5.78	5.99
11	1	−1	0	1.68	1.69
12	1	0	−1	1.96	1.89
13	1	1	0	3.12	3.28
14	0	−1	−1	2.67	2.73
15	0	0	0	6.22	5.99
16	−1	0	1	4.22	4.29
17	−1	1	0	2.94	2.93

**Table 7:** Analysis of variance of BBD.

Source	Freedom	Sum of Squares	Mean Square	*F* Value	Prob > *F*
Model	9	41.12	4.57	104.15	<0.0001 ***
X_1_	1	2.33	2.33	53.18	0.0002 ***
X_3_	1	0.053	0.053	1.20	0.3088
X_4_	1	3.45	3.45	78.54	<0.0001 ***
X_1_X_3_	1	2.03	2.03	46.29	0.0003 ***
X_1_X_4_	1	3.48	3.48	79.29	<0.0001 ***
X_3_X_4_	1	1.35	1.35	30.67	0.0009 ***
X_12_	1	3.53	3.53	80.36	<0.0001 ***
X_32_	1	17.74	17.74	404.35	<0.0001 ***
X_42_	1	4.66	4.66	106.32	<0.0001 ***
Residual	7	0.31	0.044		
Lack of Fit	3	0.10	0.033	0.64	0.6258
Pure Error	4	0.21	0.052		
C. Total	16	41.43			
Standard deviation		0.21	R-squared		0.9926
Mean		4.10	Adjusted R-squared		0.9831
Coefficient of variation (C.V.%)		5.11	Predicted R-squared		0.9535
PRESS		1.92	Adequate precision		26.7760

*** indicates a significant difference at *p* < 0.001 level.

## Data Availability

Not applicable.

## Supplementary Materials

The following supporting information can be downloaded at: https://www.mdpi.com/article/10.3390/microorganisms10091854/s1.
