# Supplementary material for: Response Surface Methodology (RSM) Mediated Optimization of Medium Components for Mycelial Growth and Metabolites Production of Streptomyces alfalfae XN-04
Source: Microorganisms. 2022 Sep 16;10(9):1854. doi: 10.3390/microorganisms10091854 (PMC9501596; doi:10.3390/microorganisms10091854)
Supplement: Supplementary file 1 [file microorganisms-10-01854-s001.zip › microorganisms-1886883-SI.pdf]

Supplementary Material

1.1 Supplementary Figures

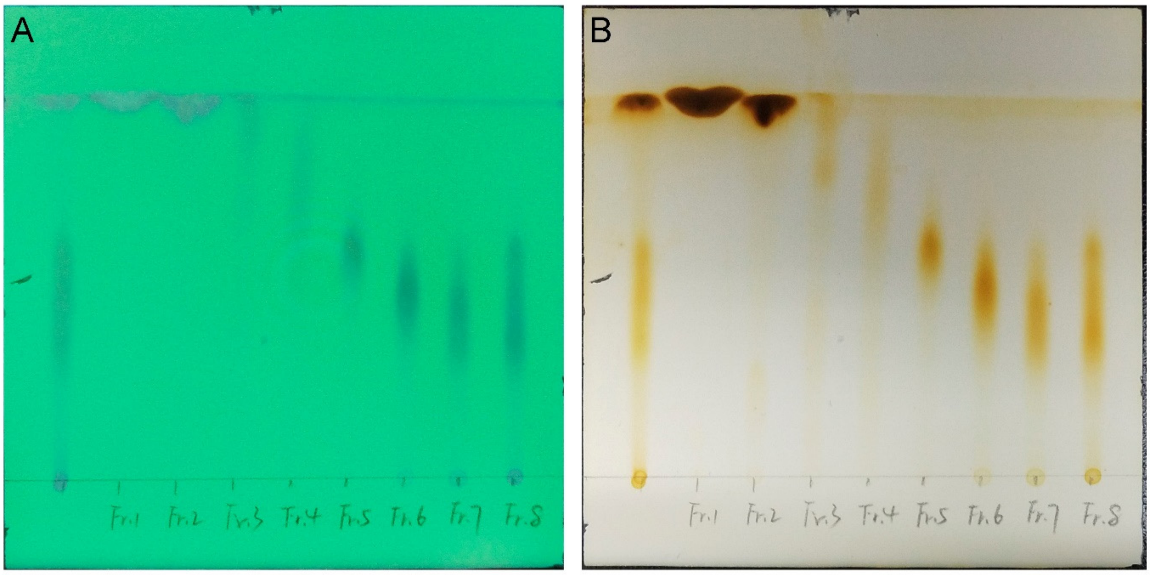

Supplementary Figure S1. Thin layer chromatography (TLC) analysis of the MeOH extract from *S. alfalfae* XN-04. Frs.1-8 were purified by silica gel column chromatography. (A) Under ultraviolet light of 254 nm (UV_{254}); (B) With iodine.

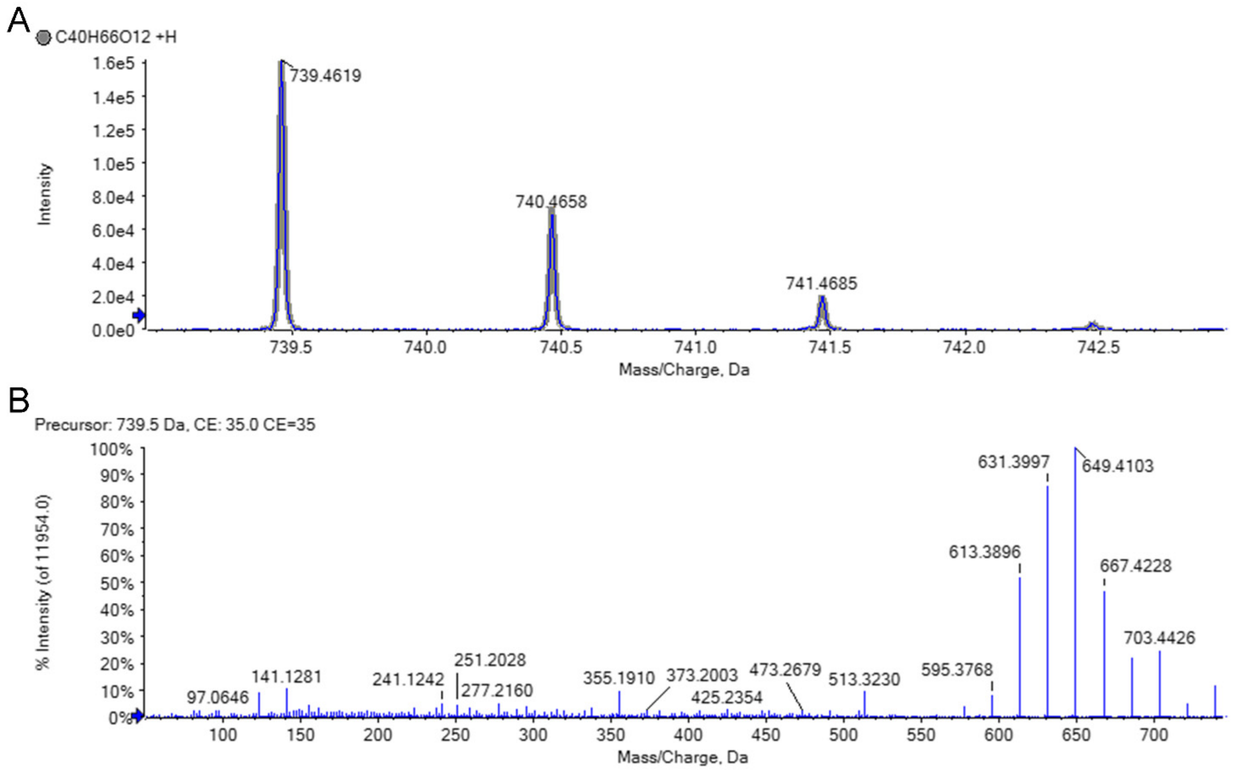

Supplementary Figure S2. Mass spectrum of the compound exhibiting antifungal activity isolated from *S. alfalfae* XN-04. (A) Mass spectrum of the peak at m/z 739.4619. (B) Tandem mass spectrometry (MS/MS) spectrum of the peak at m/z 739.4619.

1.2 Supplementary Tables

Table S1. Ten fractions purified by silica gel column chromatography.

Fractions	Weight (mg)	Inhibition radius (mm)
Fr.1	1174	0
Fr.2	5631	0
Fr.3	110	0
Fr.4	176	1
Fr.5	672	14
Fr.6	755	10
Fr.7	459	10
Fr.8	860	12
Fr.9	165	3
Fr.10	1028	0

Table S2. ^{1}H and ^{13}C NMR data of component 1 (600 MHz).

Position	δ C	δ H
1	170.1 C	— —
2	127.5 C	— —
3	140.6 CH	7.26 (1H, dd, 11.1, 1.3)
4	128.3 CH	6.60 (1H, dd, 14.6, 11.1)
5	141.8 CH	6.66 (1H, dd, 14.6, 10.5)
6	133.2 CH	6.41 (1H, dd, 14.8, 10.5)
7	138.5 CH	6.48 (1H, dd, 14.8, 10.6)
8	132.0 CH	6.25 (1H, dd, 14.8, 10.6)
9	137.0 CH	6.36 (1H, dd, 14.8, 10.6)
10	133.5 CH	6.19 (1H, dd, 14.8, 10.6)
11	135.0 CH	5.83 (1H, m)
12	40.5 CH	2.40 (1H, m)<br>2.22 (1H, m)
13	69.0 CH	4.03 (1H, overlap)
14	41.9 CH_2	1.91 (1H, m)<br>1.15 (1H, m)
15	65.1 CH	4.06 (1H, overlap)
16	45.6 CH_2	1.97 (1H, m)<br>1.24 (1H, m)
17	98.9 C	— —
18	49.6 CH_2	1.80 (1H, overlap)<br>1.69 (1H, overlap)
19	66.1 CH	4.31 (1H, overlap)
20	46.1 CH_2	1.69 (1H, overlap)<br>1.45 (1H, overlap)
21	66.2 CH	4.06 (1H, overlap)
22	46.4 CH_2	1.51 (1H, overlap)<br>1.39 (1H, overlap)
23	68.8 CH	3.96 (1H, overlap)
24	46.5 CH_2	1.53 (1H, overlap)<br>1.45 (1H, overlap)
25	68.3 CH	4.03 (1H, overlap)
26	45.9 CH_2	1.45 (1H, overlap)<br>1.39 (1H, overlap)
27	68.8 CH	3.96 (1H, overlap)
28	45.6 CH_2	1.51 (1H, overlap)<br>1.37 (1H, overlap)
29	72.3 CH	3.96 (1H, overlap)
30	44.1 CH_2	1.51 (1H, overlap)<br>1.37 (1H, overlap)
31	72.6 CH	3.62 (1H, m)
32	36.2 CH_2	1.57 (1H, overlap)<br>1.45 (1H, overlap)
33	31.2 CH_2	1.21 (1H, m)<br>1.08 (1H, overlap)
34	35.7 CH	1.80 (1H, overlap)
35	81.4 CH	4.81 (1H, dd, 10.0, 2.4)

36	30.9 CH	1.97 (1H, m)
37	20.3 CH_3	0.88 (3H, t, 6.7)
38	19.2 CH_3	0.93 (3H, t, 6.7)
2-Me	12.9 CH_3	1.98 s
38-Me	14.4 CH_3	0.96 (3H, t, 6.8)
